# Associations Between Physical Activity Patterns and Cardiovascular Events and Risk Factors: Cross-Sectional and Prospective Studies

**DOI:** 10.1016/j.jacadv.2024.101324

**Published:** 2024-10-09

**Authors:** Fabrizio Cominetti, Julien Vaucher, Pedro Marques-Vidal, Vanessa Kraege

**Affiliations:** a Internal Medicine Division, Department of Medicine, Lausanne University Hospital and University of Lausanne, Lausanne, Switzerland; b Division of Internal Medicine, Department of Internal Medicine and Specialties, Hospital and University of Fribourg, Fribourg, Switzerland; c Medical and Innovation and Clinical Research Directorates, Lausanne University Hospital and University of Lausanne, Lausanne, Switzerland

**Keywords:** cardiovascular, chronotype, chronoactivity, epidemiology, physical activity

## Abstract

**Background:**

Moderate-intensity physical activity (PA) is recommended for health benefits, but optimal PA timing regarding cardiovascular disease (CVD) is debated.

**Objectives:**

The authors assessed the impact of differing PA patterns on CVD risk factors and outcomes.

**Methods:**

Data from 2 surveys (S1 and S2) of the CoLaus-PsyCoLaus study (2,465 and 1,692 participants, respectively; 55.3% [54.3%] females; mean age 61.2 ± 9.7 years [64.4 ± 9.5]), conducted in Lausanne, Switzerland. PA was assessed using a wrist-worn accelerometer, and PA patterns were assessed using K-means clustering.

**Results:**

Morning PA was positively associated with hypertension (multivariable-adjusted OR: 1.36 [95% CI: 1.00-1.84]) in S1, similar trend in S2. No significant association was found between PA clusters and total, HDL-, and LDL-cholesterol or triglycerides. Morning PA was positively associated with hypolipidemic drug treatment: 1.88 (1.07-3.30) in S2. Evenly distributed daily PA was positively associated with diabetes: 1.82 (95% CI: 1.06-3.12) in S2, with a similar trend in S1. In the outcome analysis, the early morning PA cluster (7 am-12 am) and the evenly distributed daily PA cluster led to a higher risk of CVD events (HR: 3.33 [95% CI: 1.08-10.3] and 3.16 [95% CI: 1.04-9.57], respectively).

**Conclusions:**

In a population-based study, we observed a higher risk for cardiovascular events in participants whose daily PA occurred predominantly in the early morning (7 am-12 am) or was evenly distributed throughout the day. No PA pattern was consistently associated with hypertension, blood lipids, or diabetes markers.

International guidelines recommend 150 to 300 minutes of moderate-intensity physical activity (PA) per week to maintain good health.^1^ Research has long focused on frequency, intensity, and total amount of PA, but the optimal timing of PA remains unclear regarding general health benefits.^2^

A few studies have focused on health and cardiometabolic effects of PA timing. A systematic review in 2020 showed no conclusive evidence favoring PA at a certain time of day for effect on adiposity markers and cardiometabolic biomarkers.^2^ Still, a recent study using accelerometer-based PA evaluation in the UK Biobank showed an association between morning PA and lower risk of cardiovascular disease (CVD), irrespective of daily total PA.^3^ Another study on the same cohort assessed the effects of the timing of moderate to vigorous PA (MVPA) on all-cause and specific mortality; the authors concluded that CVD mortality was lower in participants whose MVPA was higher at midday and in the afternoon.^4^ Indeed, timing of PA and its concordance with chronotype appear to be an important factor in CVD mortality risk, with early morning and nighttime PA being less favorable than midday activity.^5^

In a post-hoc analysis of a randomized controlled trial, Albalak et al showed an association between increased morning PA and several metabolic parameters, including body mass index (BMI).^6^ A cross-sectional analysis of a diabetic population showed sex differences, morning MVPA being associated with better cardiorespiratory fitness in males, whereas evening MVPA was associated with better cardiorespiratory fitness in females.^7^ In a Dutch cohort, insulin resistance was reduced in groups with higher afternoon and evening MVPA compared to a group with evenly distributed MVPA throughout the day.^8^ Similarly, evening PA was associated with better blood pressure outcomes in Japanese male workers,^9^ and with better health-related fitness in Finnish workers.^10^ Still, the analysis of existing data is complicated as most authors used different methods to quantify PA and its timing.

Hence, our study aimed to assess the impact of the time of day of accelerometery-assessed PA on cardiometabolic parameters (BMI, waist circumference, blood pressure, blood glucose levels, lipid levels) using data from a population-based study.

## Methods

### Participants

The study used data from the CoLaus-PsyCoLaus study, a prospective study conducted in the population of Lausanne, Switzerland. Details of the recruitment procedure have been reported previously.^11^ Three follow-ups were performed in 2009 to 2012, 2014 to 2017, and 2018 to 2021. They included 5,064 (75.2%), 4,881 (72.5%), and 3,751 (55.7%), respectively, of the 6,733 initial participants. Objectively assessed PA was conducted in the second and third follow-ups; hence, data from those 2 follow-ups was used in this study. For simplicity, the second and third follow-ups will be named survey 1 (S1) and survey 2 (S2), respectively. Each survey was considered and analyzed independently in both cross-sectional analyses. In other words, the cohort’s population was cross-sectionally analyzed at 2 distinct moments, in the first survey and then 3.8 years (median) later in the second. For the outcome analysis, S1 participants were studied, as they had 2 consecutive PA assessments (in second and third follow-up).

### Physical activity

PA was objectively assessed using a wrist-worn triaxial accelerometer (GENEActiv, Activinsights Ltd, www.activinsights.com). These devices are the same as those used in the UK biobank study,^12^ weigh 16 g and allow continuous monitoring of PA for a maximum of 45 days. The devices were preprogrammed with a 50 Hz sampling frequency and subsequently attached to the participants’ right wrist. Participants were requested to wear the device continuously for 14 days in their free-living conditions. Raw accelerometry data were downloaded using the GENEActiv software version 2.9 (GENEActiv, Activinsights Ltd) and transformed into 1-minute epoch files using the GENEActiv MACRO,^6^^,^^13^ which also computes the vector magnitude (vectogram).

Data from days 2 to 8 of recording was used, as data from day 1 is not representative while wearers get used to the accelerometer. For each participant and day of recording, the average vectogram for each hour was computed to assess PA. Then, for each individual, the daily hourly values of PA were standardized by the 7-day mean of total daily PA as follows:

$${PAstd}_{i}=\frac{{\;\bar{PA}}_{i}}{\bar{{PA}_{24}}},where\;i=1\;to\;24$$

The standardized PA for hour *i* is the ratio of the average PA for time *i* divided by the average of the 24-hour PA. For example, hour *i* = 1 corresponds to the time between 0 and 59 minutes am. The 24 standardized hourly values were then grouped into clusters using K-means clustering analysis as performed by Albalak et al^3^ to obtain standardized relative PA profiles. We determined the number of 4 clusters with proportional reduction in error testing. As each cluster has a definite relative PA pattern, they will be referred to as “PA timing cluster.” This number also allowed comparison with previously published results.

### Cardiometabolic risk factors

Body weight and height were measured with participants barefoot and in light indoor clothes. Body weight was measured in kilograms to the nearest 100 g using a Seca scale. Height was measured to the nearest 5 mm using a Seca height gauge. BMI was categorized as normal (<25 kg/m^2^), overweight (≥25- <30 kg/m^2^), and obesity (≥30 kg/m^2^).

Waist circumference was measured midway between the lowest rib and the iliac crest using a nonstretchable tape, and the average of 2 measurements was taken. Abdominal obesity was defined as a waist circumference >102 cm (males) or >88 cm (females).

Blood pressure (BP) was measured using an Omron HEM-907 automated oscillometric sphygmomanometer after at least a 10-minute rest in a seated position, and the average of the last 2 measurements was used. Hypertension was defined by a systolic blood pressure (SBP) ≥140 mm Hg or a diastolic blood pressure (DBP) ≥90 mm Hg or presence of antihypertensive drug treatment.

Biological assays were performed by the Centre Hospitalier Universitaire Vaudois Clinical Laboratory on fresh blood samples within 2 hours of blood collection. Fasting plasma glucose was assessed by glucose hexokinase. Diabetes was defined as the presence of antidiabetic treatment and/or a fasting blood glucose ≥7 mmol/L or glycated hemoglobin level (HbA_1_c) above 48 mmol/mol.

Total cholesterol was assessed by cholesterol oxidase phenol 4-aminoantipyrine peroxidase; high-density lipoprotein-cholesterol was assessed by cholesterol oxidase phenol 4-aminoantipyrine peroxidase + polyethylene glycol + cyclodextrin. Triglycerides were assessed by glycerol phosphate oxidase phenol 4-aminoantipyrine peroxidase; low-density lipoprotein-cholesterol levels were computed using the Friedewald formula.

### Cardiovascular outcomes

During the follow-up period, first incident CVD events and deaths were prospectively collected and independently adjudicated according to established recommendations and similar definitions detailed elsewhere.^11^ Details of the adjudication procedure are provided in the Supplemental Appendix.

### Covariates

Socio-economic and lifestyle data were collected by questionnaire. Education was categorized as mandatory education, apprenticeship, high school, and university education. Smoking was categorized as never, former, and current. Medications were collected and classified according to the World Health Organization ATC criteria. Alcohol consumption was categorized as nondrinker vs number of units per week (1-13, 14-27, 28 or more). Morning/evening chronotype was assessed by questionnaire.

### Inclusion and exclusion criteria

For the cross-sectional analyses, participants were excluded if they had: 1) no or <1 week of valid PA data; 2) presented with previous CVD; or 3) missing data for outcomes or covariates. For the outcome analysis, participants were also excluded if they; 4) already presented with CVD; or 5) had no follow-up after the first survey.

### Statistical analysis

Statistical analyses were conducted using Stata version 18.0 for Windows (Stata Corp). Participant characteristics were expressed as number (percentage) for categorical variables or as mean ± SD for continuous variables. Between-group comparisons were performed using chi-square or Fisher’s exact test for categorical variables and a Student’s *t*-test, analysis of variance, or Kruskal-Wallis test for continuous variables.

Cross-sectional multivariable analyses were conducted using logistic regression for categorical outcomes (hypertension, hypolipidemic drug treatment, diabetes [yes, no]), and results were expressed as OR (95% CI). For continuous outcomes, multivariable analyses were conducted using analysis of variance, and results were expressed as adjusted mean ± SEM. Multivariable analyses were adjusted on sex, age, education, smoking, and alcohol consumption for all outcomes. For hypertension, diabetes and hypolipidemic drug treatment, further adjustment on BMI categories was performed. For blood pressure, glycemia, and lipids, further adjustment on antihypertensive, antidiabetic, and hypolipidemic drug treatment was performed.

For the outcome analysis, the incidence of CVD events, CVD-specific, and overall mortality were computed for each PA timing cluster. Unadjusted and adjusted analyses were performed using Cox regression for overall mortality and incident fatal and nonfatal CVD events. Proportional hazards assumption was tested using the Stata command estat phtest and no deviations were found. Results were expressed as HR (95% CI). Multivariable analyses were performed adjusting for sex, age, education, smoking, alcohol consumption, and BMI categories in a first adjusted model. In the second model, we conducted supplementary adjustments for hypertension, diabetes, and hypolipidemic drug treatment. A Fine-Gray model using non-CVD death as competing risk was also applied, adjusting for the same variables as before (third model).

Furthermore, analysis of each PA timing cluster was also stratified by age (<65 or >65 years of age) and PA level (less or more active groups: a participant is categorized in the more active group if its mean PA vector over the entire study period is above the sample’s median). A second analysis investigated the association between mean hourly physical activity of total study population and cardiovascular disease events using Cox-proportional hazard model, adjusted for age and sex. Finally, we performed inverse probability weighting in the outcome analysis. Statistical significance was considered for a 2-sided test with *P* < 0.05.

### Ethical statement

The institutional Ethics Committee of the University of Lausanne, which afterward became the Ethics Commission of Canton Vaud, approved the baseline CoLaus study (reference 16/03). The approval was renewed for the first (reference 33/09), the second (reference 26/14), and the third (reference PB_2018-00040) follow-ups. The approval for the entire CoLaus|PsyCoLaus study was confirmed in 2021 (reference PB_2018-00038, 239/09). The full decisions of the Ethics Committee of Canton Vaud can be obtained from the authors upon request. The study was performed in agreement with the Helsinki Declaration and its former amendments and in accordance with the applicable Swiss legislation. All participants gave their signed informed consent before entering the study.

## Results

### Study samples

**Figure 1:**
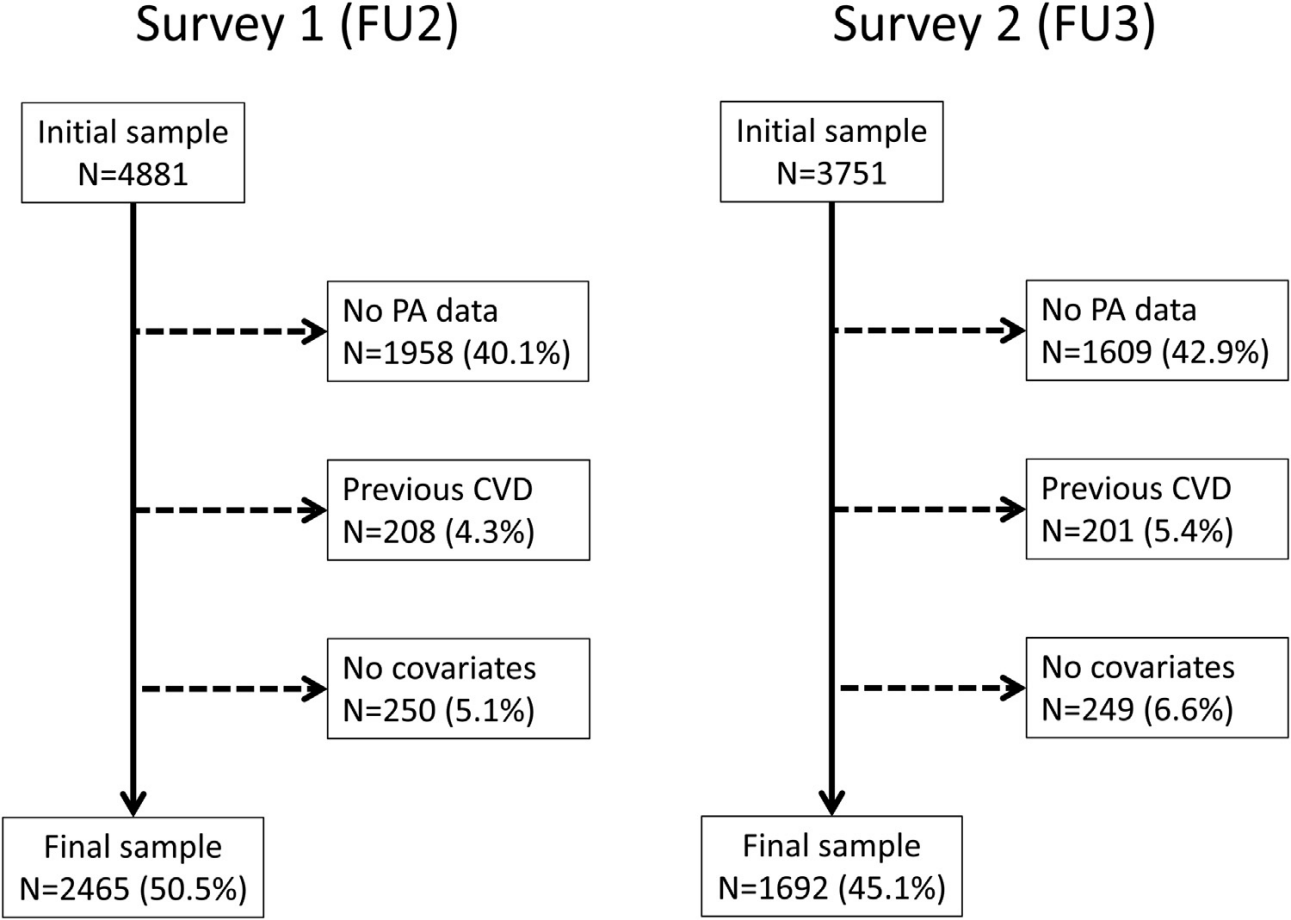
**Selection of Participants for Surveys 1 and 2 Follow-Ups, CoLaus-PsyCoLaus Study, Lausanne, Switzerland** CVD = cardiovascular disease; FU2 = second follow-up; FU3 = third follow up; PA = physical activity.

Of the initial 4,881 and 3,751 participants in the first and second surveys, 2,465 (50.5%) and 1,692 (45.1%), respectively, were retained for analysis. The reasons for exclusion are indicated in Figure 1 and the comparison between included and excluded participants is provided in Supplemental Table 1. Excluded participants were older, less well educated, with a different chronotype, more frequently teetotalers, had higher levels of BMI, waist circumference, fasting glucose and insulin, lipids, and systolic blood pressure, and were more likely to have an antidiabetic, antihypertensive, or cholesterol-lowering medication.

### Characteristics of physical activity clusters

**Figure 2:**
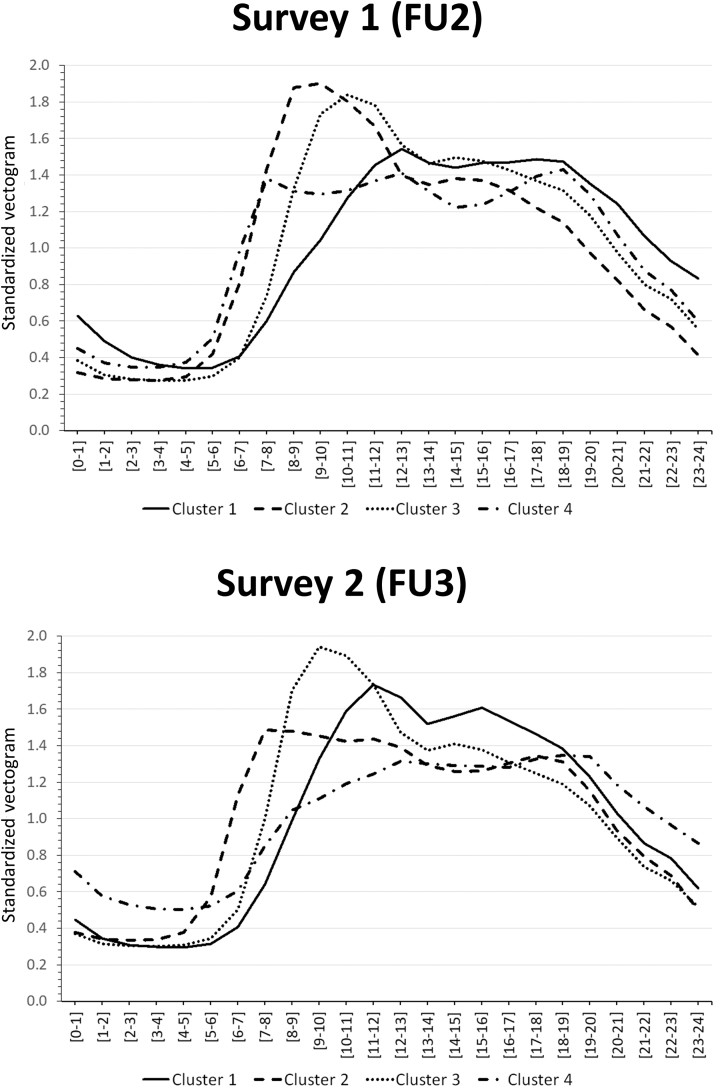
**Physical Activity Timing Clusters for Survey 1 and 2, CoLaus-PsyCoLaus Study, Lausanne, Switzerland** Patterns of standardized vectogram showing mean relative PA of each cluster in the first and second surveys. Cluster 1: PA peaks between 10 am and 2 pm. Cluster 2: PA peaks between 7 am and 12 am. Cluster 3: PA peaks between 8 am and 1 pm. Cluster 4 demonstrates no clear peak of PA. FU2 = second follow-up; FU3 = third follow-up; PA = physical activity.

Within each follow-up, each participant was classified into a single cluster. The number of clusters was selected according to the proportional reduction in error. For the first survey, the number of clusters was 4, and for the second survey, it could be either 4 or 5 (Supplemental Figures 1 and 2). We selected 4 clusters as it agreed with the results and also allowed comparison with the literature. The PA clusters were quite similar between surveys (Figure 2). In cluster 1, PA peaked between 10 am and 2 pm; in cluster 2, PA peaked between 7 am and 12 am; in cluster 3, PA peaked between 8 am and 1 pm and in cluster 4, no clear peak of PA was observed.

The sociodemographic characteristics of the PA clusters according to survey are summarized in Supplemental Table 2. In both surveys, clusters differed regarding age, sex, educational level, and smoking status distribution. In S2 (third follow-up), clusters also differed significantly regarding BMI, waist circumference, and sleep chronotype. Cluster 4 was the youngest, with less females, more educated participants, and a higher prevalence of current smokers.

### Association of physical activity timing clusters with cardiometabolic risk factors

The unadjusted and adjusted analysis of cardiometabolic markers according to PA timing clusters is provided in Tables 1 and 2 for S1 and S2, respectively. After multivariable adjustment, no association was found between clusters and SBP or DBP. Cluster 2 was positively associated with hypertension in S1, with a nonsignificant trend in S2 (Tables 1 and 2). Regarding lipids, no association was found between clusters and HDL cholesterol or triglycerides. Cluster 3 had the highest total and LDL cholesterol levels in S1, but this was not confirmed in S2. Cluster 2 was positively associated with hypolipidemic drug treatment in S2, but this was not confirmed in S1 (Tables 1 and 2). Regarding diabetes, no association was found between clusters and glucose, HbA_1_c or insulin levels. Cluster 4 was positively associated with diabetes in S2, but this was not confirmed in S1 (Tables 1 and 2).

**Table 1:** Cardiometabolic Risk Factors by Physical Activity Timing Clusters, Survey 1 (Second Follow-Up, 2014-2017), CoLaus-PsyCoLaus Study, Lausanne, Switzerland

	Cluster Number				
	1 (n = 481)	2 (n = 453)	3 (n = 790)	4 (n = 741)	*P* Value
Blood pressure markers					
SBP (mm Hg)					
Unadjusted	124 ± 17	128 ± 18	127 ± 18	123 ± 17	<0.001
Adjusted	124 ± 1	126 ± 1	126 ± 1	125 ± 1	0.188
DBP (mm Hg)					
Unadjusted	77 ± 11	77 ± 10	78 ± 11	78 ± 11	0.735
Adjusted	77 ± 1	78 ± 1	78 ± 1	77 ± 1	0.448
Hypertension					
Unadjusted	171 (35.6)	223 (49.2)	338 (42.8)	260 (35.1)	<0.001
Adjusted	1.00 (reference)	**1.36 (1.00-1.84)**	1.14 (0.87-1.49)	1.14 (0.87-1.50)	
Lipid markers					
Total cholesterol (mmol/L)					
Unadjusted	5.4 ± 0.9	5.4 ± 1.0	5.5 ± 1.0	5.3 ± 0.9	<0.001
Adjusted	5.34 ± 0.04	5.36 ± 0.05	5.50 ± 0.04	5.37 ± 0.04	0.018
HDL cholesterol (mmol/L)					
Unadjusted	1.6 ± 0.5	1.7 ± 0.5	1.6 ± 0.4	1.5 ± 0.5	<0.001
Adjusted	1.62 ± 0.02	1.65 ± 0.02	1.60 ± 0.01	1.59 ± 0.02	0.126
LDL cholesterol (mmol/L)					
Unadjusted	3.2 ± 0.9	3.2 ± 0.9	3.3 ± 0.9	3.2 ± 0.8	0.007
Adjusted	3.14 ± 0.04	3.17 ± 0.04	3.30 ± 0.03^a^	3.21 ± 0.03	0.008
Triglycerides (mmol/L)					
Unadjusted	1.3 ± 1.0	1.2 ± 0.8	1.3 ± 1.1	1.3 ± 1.1	0.597^a^
Adjusted	1.30 ± 0.05	1.22 ± 0.05	1.33 ± 0.04	1.27 ± 0.04	0.057^a^
Hypolipidemic drug treatment					
Unadjusted	53 (11.3)	78 (17.9)	124 (16.2)	84 (11.7)	0.002
Adjusted	1.00 (reference)	1.13 (0.73-1.73)	1.26 (0.86-1.86)	1.44 (0.96-2.15)	
Diabetes markers					
Glucose (mmol/L)					
Unadjusted	5.3 ± 0.9	5.4 ± 1.1	5.4 ± 0.9	5.4 ± 1.0	0.573
Adjusted	5.36 ± 0.04	5.41 ± 0.04	5.37 ± 0.03	5.38 ± 0.03	0.865
HbA_1_c (mmol/mol)					
Unadjusted	37.4 (6.3)	38.0 (6.7)	37.9 (5.7)	37.1 (6.0)	0.036
Adjusted	37.6 ± 0.2	37.6 ± 0.3	37.5 ± 0.2	37.7 ± 0.2	0.923
Insulin (μIU/mL)					
Unadjusted	8.8 ± 5.3	9.3 ± 6.5	9.1 ± 5.9	9.0 ± 6.8	0.618^a^
Adjusted	8.9 ± 0.3	9.2 ± 0.3	9.0 ± 0.2	9.3 ± 0.2	0.821^a^
Diabetes^b^					
Unadjusted	33 (6.9)	37 (8.2)	66 (8.4)	54 (7.3)	0.737
Adjusted	1.00 (reference)	0.84 (0.48-1.46)	1.04 (0.64-1.71)	1.19 (0.72-1.96)	

Values are mean ± SD, n (%), or OR (95% CI).

ANOVA = analysis of variance; BMI = body mass index; DBP = diastolic blood pressure; HDL cholesterol = high density lipoprotein cholesterol; LDL cholesterol = low-density lipoprotein cholesterol; SBP = systolic blood pressure.

a Based on log-transformed data.

b Defined as a fasting plasma glucose ≥7 mmol/L and/or presence of antidiabetic drug treatment. Between-group comparisons were performed using Pearson chi-squared test for categorical variables and analysis of variance for continuous variables. Statistically significant (*P* < 0.05) ORs are indicated in **bold**. Multivariable analysis is performed using logistic regression for categorical variables and ANOVA for continuous variables. For continuous variables, post-hoc bivariate comparisons were performed using Scheffe’s method: ^a^comparison of clusters 2, 3, and 4 to cluster 1, ^b^comparison of clusters 3 and 4 to cluster 2, and ^c^comparison of cluster 4 to cluster 3; presence of the subscript indicates that clusters differ at *P* < 0.05. All multivariable models adjusted for sex (male, female), age (continuous), educational level (basic, apprenticeship, secondary, high school, and university), alcohol consumption (none, 1-13, 14-27, and 27+ per week), smoking categories (never, former, and current), and BMI categories (normal, overweight, and obese). For blood pressure levels, a further adjustment on antihypertensive drug treatment (yes, no) was performed; for lipid levels, a further adjustment on hypolipidemic drug treatment (yes, no) was performed; and for diabetes markers, a further adjustment on antidiabetic drug treatment (yes, no) was performed.

**Table 2:** Cardiometabolic Risk Factors by Physical Activity Timing Clusters, Survey 2 (Third Follow-Up, 2018-2021), CoLaus-PsyCoLaus Study, Lausanne, Switzerland

	Cluster Number				
	1 (n = 458)	2 (n = 413)	3 (n = 480)	4 (n = 341)	*P* Value
Blood pressure markers					
SBP (mm Hg)					
Unadjusted	128 ± 17	128 ± 18	129 ± 18	126 ± 17	0.047
Adjusted	128 ± 1	128 ± 1	127 ± 1	126 ± 1	0.245
DBP (mm Hg)					
Unadjusted	78 ± 10	78 ± 11	77 ± 10	78 ± 11	0.769
Adjusted	78 ± 1	77 ± 1	78 ± 1	77 ± 1	0.268
Hypertension					
Unadjusted	199 (43.5)	185 (44.8)	239 (49.8)	146 (42.8)	0.144
Adjusted	1.00 (reference)	1.14 (0.84-1.54)	0.99 (0.74-1.33)	1.01 (0.73-1.39)	
Lipid markers					
Total cholesterol (mmol/L)					
Unadjusted	5.4 ± 0.9	5.2 ± 0.9	5.3 ± 1.0	5.2 ± 1.0	0.049
Adjusted	5.46 ± 0.08	5.32 ± 0.08	5.34 ± 0.07	5.30 ± 0.09	0.496
HDL cholesterol (mmol/L)					
Unadjusted	1.6 ± 0.4	1.6 ± 0.5	1.6 ± 0.4	1.5 ± 0.4	<0.001
Adjusted	1.47 ± 0.03	1.53 ± 0.03	1.51 ± 0.03	1.47 ± 0.04	0.450
LDL cholesterol (mmol/L)					
Unadjusted	3.2 ± 0.8	3.1 ± 0.8	3.1 ± 0.9	3.1 ± 0.9	0.234
Adjusted	3.29 ± 0.07	3.17 ± 0.08	3.17 ± 0.07	3.14 ± 0.08	0.431
Triglycerides (mmol/L)					
Unadjusted	1.3 ± 0.7	1.4 ± 1.2	1.3 ± 0.9	1.4 ± 1.0	0.558^a^
Adjusted	1.53 ± 0.08	1.50 ± 0.09	1.44 ± 0.07	1.54 ± 0.09	0.387^a^
Hypolipidemic drug treatment					
Unadjusted	49 (38.3)	56 (47.9)	86 (54.1)	48 (49.0)	0.065
Adjusted	1.00 (reference)	**1.88 (1.07-3.30)**	1.33 (0.78-2.25)	1.39 (0.77-2.53)	
Diabetes markers					
Glucose (mmol/L)					
Unadjusted	5.4 ± 0.7	5.5 ± 1.1	5.5 ± 1.1	5.6 ± 1.2	0.107
Adjusted	5.50 ± 0.04	5.51 ± 0.04	5.48 ± 0.04	5.57 ± 0.05	0.577
HbA_1_c (mmol/mol)					
Unadjusted	36.6 (5.0)	36.7 (5.8)	37.4 (6.3)	37.7 (7.6)	0.021
Adjusted	36.9 ± 0.2	37.0 ± 0.3	37.0 ± 0.2	37.7 ± 0.3	0.143
Insulin (μIU/mL)					
Unadjusted	9.9 ± 8.6	9.6 ± 5.7	9.8 ± 5.6	11.3 ± 9.2	0.007^a^
Adjusted	10.3 ± 0.3	9.9 ± 0.3	9.7 ± 0.3	11.1 ± 0.4	0.054^a^
Diabetes^b^					
Unadjusted	28 (6.1)	32 (7.8)	41 (8.5)	38 (11.1)	0.080
Adjusted	1.00 (reference)	1.26 (0.73-2.19)	1.19 (0.70-2.02)	**1.82 (1.06-3.12)**	

Values are mean ± SD, n (%), or OR (95% CI).

Abbreviations as in Table 1.

a Based on log-transformed data.

b Defined as a fasting plasma glucose ≥7 mmol/L and/or presence of antidiabetic drug treatment. Between-group comparisons were performed using Pearson chi-squared test for categorical variables and analysis of variance for continuous variables. Statistically significant (*P* < 0.05) ORs are indicated in **bold**. Multivariable analysis is performed using logistic regression for categorical variables and ANOVA for continuous variables. For continuous variables, post-hoc bivariate comparisons were performed using Scheffe’s method: ^a^comparison of clusters 2, 3, and 4 to cluster 1, ^b^comparison of clusters 3 and 4 to cluster 2, and ^c^comparison of cluster 4 to cluster 3; presence of the subscript indicates that clusters differ at *P* < 0.05. All multivariable models adjusted for sex (male, female), age (continuous), educational level (basic, apprenticeship, secondary, high school, and university), alcohol consumption (none, 1-13, 14-27, and 27+ per week), smoking categories (never, former, and current), and BMI categories (normal, overweight, and obese). For blood pressure levels, a further adjustment on antihypertensive drug treatment (yes, no) was performed; for lipid levels, a further adjustment on hypolipidemic drug treatment (yes, no) was performed; and for diabetes markers, a further adjustment on antidiabetic drug treatment (yes, no) was performed.

### Association of Physical Activity Timing Clusters With Incidence of Cardiovascular Events

**Figure 3:**
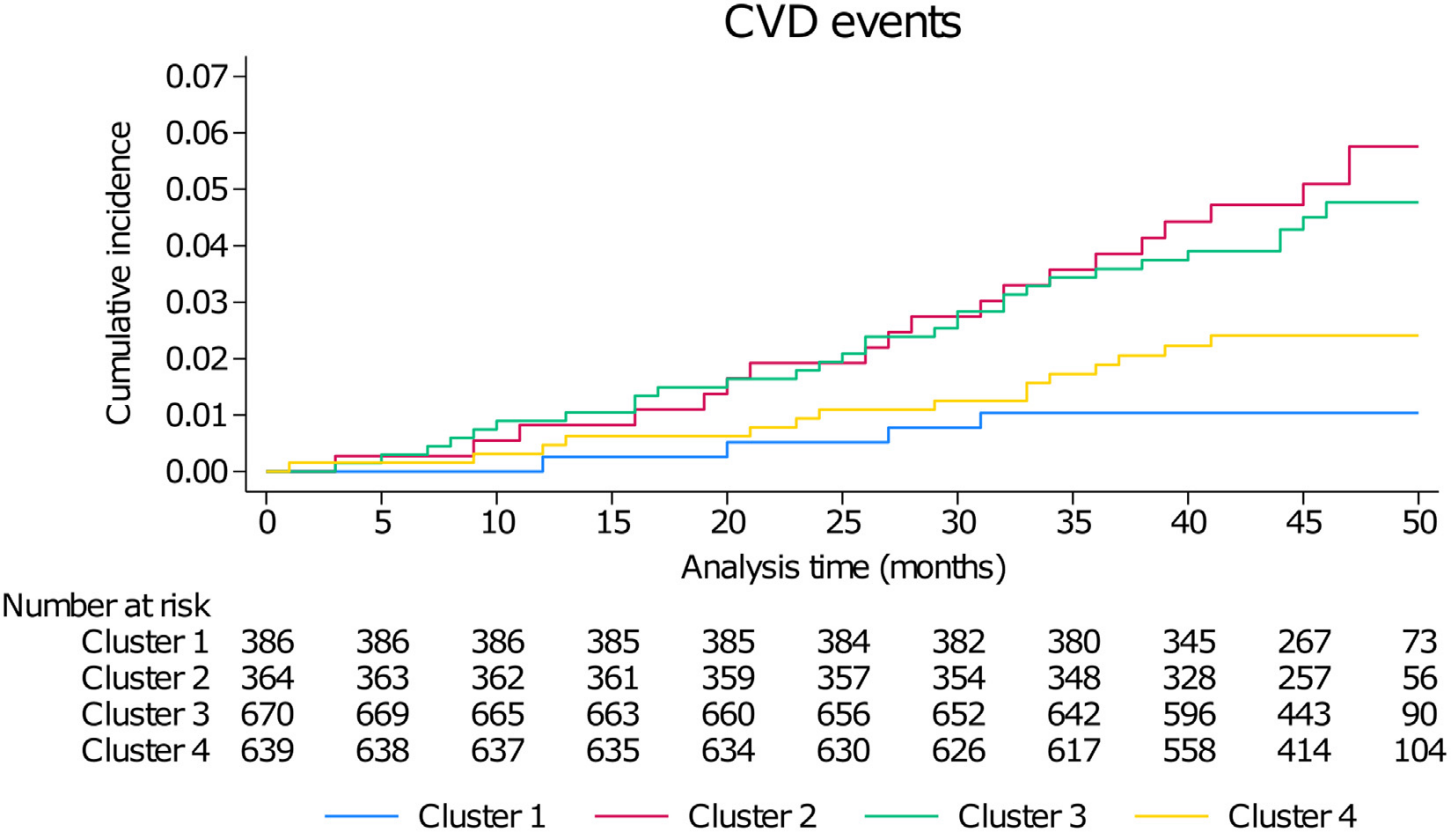
**Incidence of Cardiovascular Events in Each Physical Activity Timing Cluster, CoLaus-PsyCoLaus Study, Lausanne, Switzerland** Cumulative incidence of CVD events, univariable analysis. Cluster 1: 10 am-2 pm PA peak. Cluster 2: 7 am-12 am PA peak. Cluster 3: 8 am-1 pm PA peak. Cluster 4: no clear PA peak. CVD = cardiovascular disease; PA = physical activity.

For the outcome analysis, 2,059 participants from the first survey were included and followed for a median period of 46 months (IQR: 43-48 months). The incidence rates and association between PA timing clusters and incidence of CVD events are provided in Table 3 and the incidence curve in Figure 3. Cluster 1 had the lowest incidence rate for CVD and was selected as reference. On unadjusted analysis, participants in clusters 2 and 3 had a higher risk of developing CVD, but after multivariable adjustment (models 1, 2, and 3), clusters 2 and 4 presented with a higher risk (Table 3 and Central Illustration). The increased CVD risk in Cluster 2 and Cluster 4 persisted in the inverse probability weighted analyses, where Cluster 3 also had a higher CVD risk in the model 2 (with adjustments for hypertension, diabetes, and hypercholesterolemia) (Supplemental Table 5). Moreover, stratified analysis by age demonstrated a higher CVD risk in participants <65 years of age in Cluster 2 and Cluster 3, whereas the stratification by PA group showed that CVD risk was not increased in the more active participants of Cluster 2 and Cluster 3 (Supplemental Table 6). The second analysis of CVD risk according to mean physical activity per hour for the total study population demonstrated a nonsignificant trend for an increased risk for participants more active between 22 pm and 2 am (Supplemental Figure 3).

**Table 3:** Associations Between Physical Activity Timing Clusters and Cardiovascular Events, CoLaus-PsyCoLaus Study, Lausanne, Switzerland

	Cluster Number			
	1 (n = 386)	2 (n = 364)	3 (n = 670)	4 (n = 639)
Number of events	4	19	30	17
Incidence	0.2 (0.1-0.6)	1.2 (0.7-1.8)	1.0 (0.7-1.4)	0.6 (0.4-1.0)
Unadjusted	1.00 (reference)	**5.16 (1.76-15.2)**	**4.40 (1.55-12.5)**	2.56 (0.86-7.62)
Adjusted model 1	1.00 (reference)	**3.90 (1.29-11.8)**	2.93 (1.00-8.59)	**3.36 (1.12-10.1)**
Adjusted model 2	1.00 (reference)	**3.33 (1.08-10.3)**	2.83 (0.96-8.34)	**3.16 (1.04-9.57)**
Adjusted model 3	1.00 (reference)	**3.34 (1.11-10.1)**	2.84 (0.99-8.17)	**3.16 (1.06-9.43)**

BMI = body mass index; CVD = cardiovascular disease.

Incidence rates are expressed as rate per 1,000 person-years (and 95% CIs). For unadjusted and adjusted analysis, results are expressed as HR (95% CI). Analyses conducted using Cox regression for CVD events. Adjusted model 1: adjusted for sex (male, female), age (continuous), educational level (basic, apprenticeship, secondary, high school, and university), alcohol consumption (none, 1-13, 14-27, and 27+ per week), smoking categories (never, former, current), and BMI categories (normal, overweight, and obese). Adjusted model 2: as model 1, plus hypertension (yes, no), diabetes (yes, no), and hypolipidemic drug treatment (yes, no). Significant (*P* < 0.05) coefficients are indicated in **bold**. Adjusted model 3: Fine-Gray model, using non-CVD death as a competing risk, and adjusting as for model 2.

**Central Illustration:**
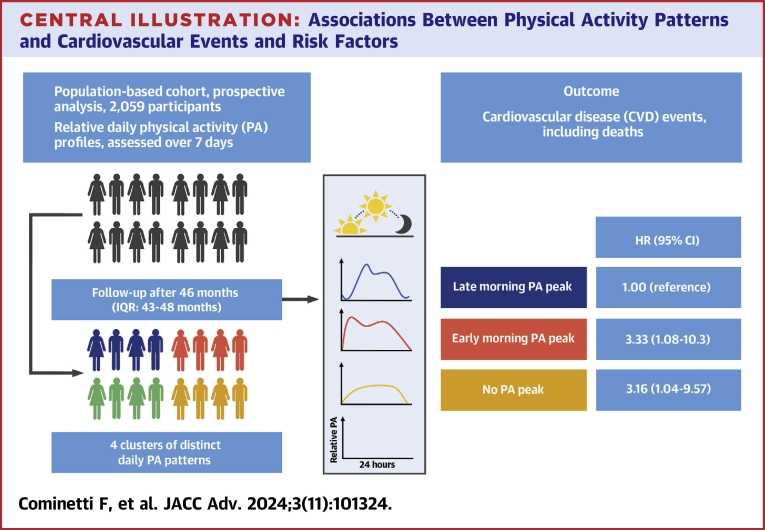
**Associations Between Physical Activity Patterns and Cardiovascular Events and Risk Factors** Participants had their physical activity objectively assessed for 7 days. They were regrouped into 4 clusters according to their relative daily physical activity patterns. Multivariable analysis after a median of 46 months of follow-up found an association of early morning PA and CVD events, also for participants with no PA peak during the day (results according to adjusted model 2 outcome analysis). CVD = cardiovascular disease; PA = physical activity.

## Discussion

### Main findings

Distinct clusters of daily PA patterns were established with K-means clustering. In the cross-sectional analysis, no consistent association was found between the 4 PA clusters and cardiovascular risk factor levels. In the outcome analysis, we found a higher risk of cardiovascular events in PA clusters with an early morning peak or no clear peak.

### Physical activity timing clusters

The patterns obtained in our study are comparable to those described by Albalak et al, who used accelerometer-based PA evaluation in the UK Biobank study.^3^ To some extent, they are also comparable with those obtained by Li et al on the same Biobank, although in our study, Cluster 4 (no clear PA peak) demonstrated a higher variability in daily PA.^14^ Cluster cross-over between S1 and S2 was observed for some participants and was more pronounced between Cluster 4 and Cluster 2, which may explain the different shape of PA patterns between follow-ups for these clusters. Nevertheless, the fact that the same clusters were obtained in different populations and settings is encouraging, as it facilitates comparisons between studies. Further, the reproducibility of those clusters suggests that they might correspond to global PA behaviors, at least in European countries.

### Association of physical activity timing clusters with cardiometabolic risk factors

No consistent differences were found between PA clusters regarding cardiometabolic risk factors.

Regarding BP and hypertension, results are conflicting regarding the best PA timing to reduce BP or prevent hypertension. Imamura et al demonstrated that evening PA was associated with better BP outcomes in Japanese male workers^9^; Li et al^14^ observed a benefit in early morning PA and early morning plus evening PA on hypertension incidence, while the Liu et al^15^ systematic review of intervention studies concluded to a benefit of morning PA on BP. The differences in SBP or DBP observed between PA groups in the UK biobank were relatively small (maximum of 6.6 mm Hg in SBP), and similar findings were reported in a systematic review.^2^^,^^3^

Regarding lipids, no conclusive trend was found in our study. This result is in keeping with published systematic reviews.^2^^,^^15^ In a review of 35 studies, Janssen et al concluded that there was no consistent evidence that PA at 1 specific period of the day provided more favorable health benefits.^2^ Similarly, in a review of 4 studies, Liu et al^15^ regrouped reported mixed results and no clear favorable moment of PA on lipid metabolism.

Regarding diabetes markers, conflicting results have been published. A post-hoc study of a randomized controlled trial showed a positive association between increased morning PA and fasting glucose, HbA_1_c, and insulin,^6^ while a Dutch cohort showed a decreased insulin resistance in groups with higher afternoon and evening PA, compared to a group with evenly distributed PA throughout the day.^8^ In Tian et al’s prospective study on type 2 diabetes risk, morning and afternoon PA were beneficial, whereas evening PA had a neutral effect.^16^ Finally, the systematic review by Janssen et al^2^ included 2 studies favoring evening PA, 1 favoring morning PA, and 4 remaining neutral regarding diabetes. Studies among diabetics also show conflicting results. A study in a Hispanic/Latino diabetic population demonstrated better HbA_1_c levels in the evening PA group.^17^ Another reported difference in the association according to sex was morning PA being more favorable for males with diabetes, while evening PA was more favorable for females with the disease.^7^

Overall, our results show no consistent associations between PA timing clusters and cardiometabolic risk factors, a finding somewhat in line with the inconclusive or conflicting results of the literature.

### Association of physical activity timing clusters with incidence of cardiovascular events

However, a difference in cardiovascular events was observed between our clusters, with early morning PA (7 am-12 am) and no clear PA peak (or evenly distributed daily PA) being associated with an increased CVD risk. The association was maintained in the sensitivity analysis according to different adjustment models. This result is part of the unequivocal body of literature on the best time of day for PA regarding CVD risk. Several studies assessed the association between timing of PA and CVD events using data from the UK Biobank. Feng et al^4^ found a decreased CVD mortality risk in participants whose PA was higher at mid-day and during the afternoon, independently of the total PA volume. Ma et al^5^ reported that mid-day PA had the lowest CVD mortality risk in individuals without pre-existing CVD. Finally, Albalak et al^3^ reported that early and late morning peaks of PA were associated with a lower CVD risk. Interestingly, different conclusions were obtained using data from the same study, which suggests that the results of the studies on the effects of timing of PA and CVD events are likely dependent on the inclusion criteria and/or methods used to identify clusters. Noteworthy, in our study, cluster cross-over between S1 and S2 was observed, being more pronounced between Cluster 4 and Cluster 2. This suggests that PA behavior changes with time, which might partly explain the conflicting results between studies. Eventually, the magnitude of the OR between clusters in our study should be cautiously interpreted, since the large CIs may indicate that our relatively small study sample could have led to a decreased precision.

### Strengths and limitations

Our study has many strengths. First, we replicated a previously used method to define clusters of PA activity, allowing an easier comparison with the literature. Secondly, our study population is drawn from a well-characterized population-based cohort. Moreover, we used objectively assessed PA data to minimize recall bias, and a potential Hawthorne effect was mitigated by excluding the first day of recording for the analyses. Finally, self-reported and clinically measured outcomes were used to study patient characteristics of each PA cluster in detail.

Still, our study also has limitations. As almost one-half of the initial sample did not have objectively assessed PA, we can hypothesize that less active participants could have been more reluctant to collect this data, leading to potential selection bias. Moreover, our study population was in a single Swiss city and mainly constituted of Caucasian subjects. Whether our results also apply in other settings should be verified. Finally, as in all studies on objectively assessed PA, we relied on PA data processed by a software, which can lead to variations in PA level estimations,^13^ and on specific methods to determine clusters of PA patterns. Nevertheless, to mitigate this effect, we used common software and replicated the clustering-methods, as stated above.^3^^,^^5^

## Conclusions

In a population-based study, we observed a higher risk for cardiovascular events in participants whose daily PA occurred predominantly in the early morning (7-12 AM) or was evenly distributed throughout the day. No association was found between PA timing and hypertension, blood lipids, or diabetes markers.

Perspectives **COMPETENCY IN MEDICAL KNOWLEDGE:** Physical activity counseling is part of daily medical routine, but it is unclear if a certain time of day is most beneficial in terms of cardiovascular health, as results are conflicting. In this study, no specific time of day seems unequivocally beneficial for cardiovascular risk-factor reduction. Further, early morning physical activity is associated with higher cardiovascular disease events.

**TRANSLATIONAL OUTLOOK:** Additional research is needed to explore if direct causality of early morning physical activity on cardiovascular disease events can be confirmed. Still, physical activity should be promoted in a way that fits a person’s preferences and capacities.

## Funding support and author disclosures

The CoLaus-PsyCoLaus study was supported by unrestricted research grants from 10.13039/100004330GlaxoSmithKline, the Faculty of Biology and Medicine of Lausanne, the 10.13039/501100001711Swiss National Science Foundation (grants 3200B0–105993, 3200B0-118308, 33CSCO-122661, 33CS30-139468, 33CS30-148401, 33CS30_177535, and 3247730_204523), and the Swiss Personalized Health Network (grant 2018DRI01). The authors have reported that they have no relationships relevant to the contents of this paper to disclose.
