# Supplementary material for: Associations Between Physical Activity Patterns and Cardiovascular Events and Risk Factors: Cross-Sectional and Prospective Studies
Source: JACC Adv. 2024 Oct 9;3(11):101324. doi: 10.1016/j.jacadv.2024.101324 (PMC11497381; doi:10.1016/j.jacadv.2024.101324)
Supplement: Supplemental data [file mmc1.docx]

### Supplemental Figure 1 – K-means clustering analysis, sum of squares plot, selection of clusters for Survey 1 (Follow-up 2, 2014-17)


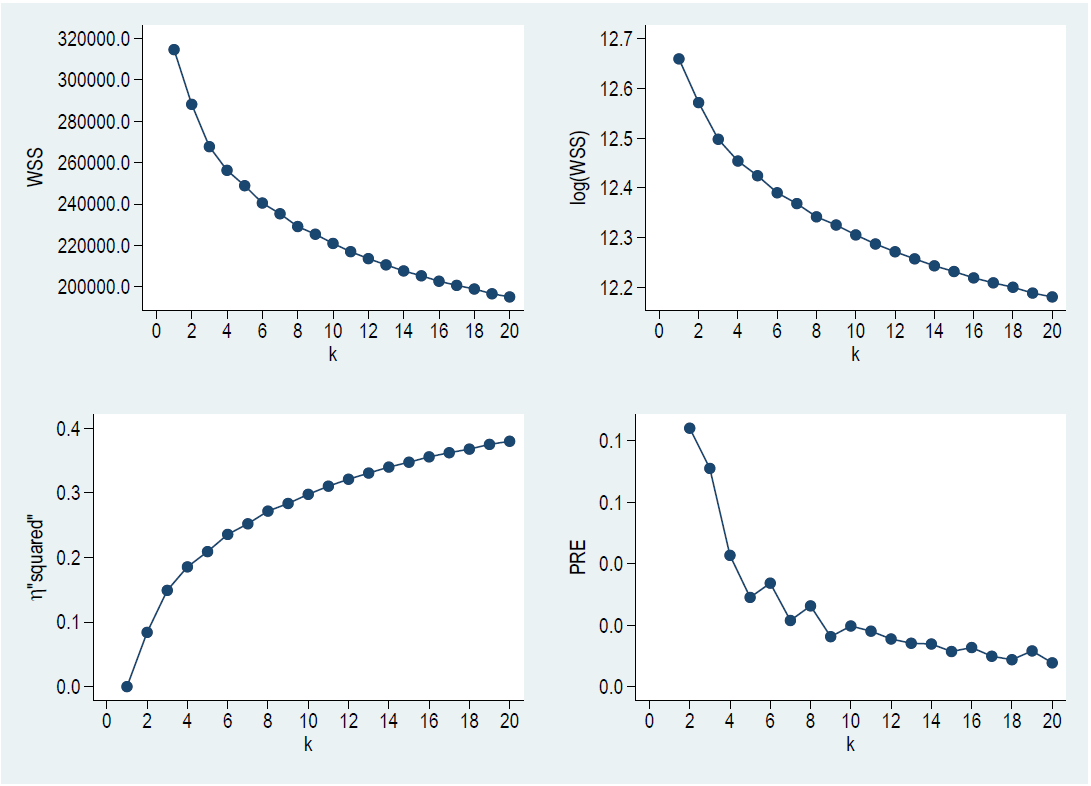


WSS, within groups sum of squares. PRE, proportional reduction of error. *η squared* measures the proportional reduction of the WSS for each cluster solution *k* compared with the total sum of squares. PRE indicates the proportional reduction of the WSS for solution with *i* clusters compared with the previous solution with *i −* 1 clusters.

### Supplemental Figure 2 – K-means clustering analysis, sum of squares plot, selection of clusters for Survey 2 (Follow-up 3, 2018-21)


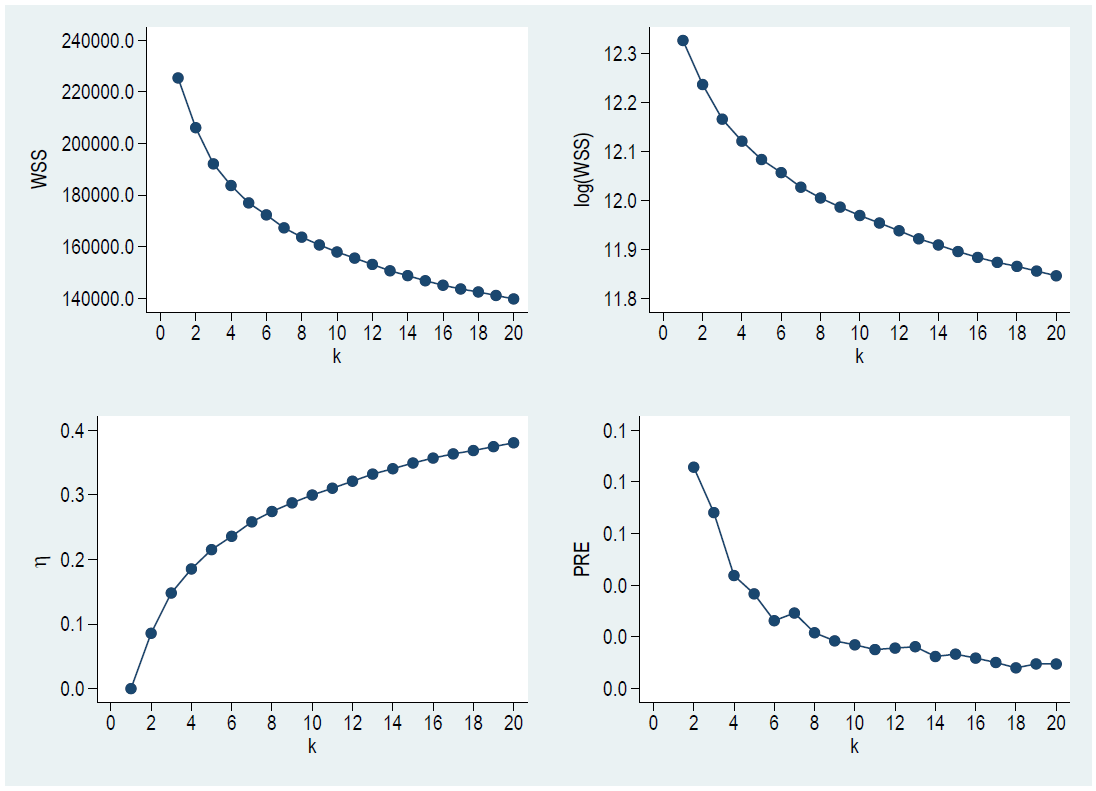


WSS, within groups sum of squares. PRE, proportional reduction of error. *η squared* measures the proportional reduction of the WSS for each solution with *k* clusters compared with the total sum of squares. PRE indicates the proportional reduction of the WSS for solution with *i* clusters compared with the previous solution with *i −* 1 clusters.

Supplemental Figure 3 – Cardiovascular disease risk according to time of day mean physical activity, CoLaus|PsyCoLaus study, Lausanne, Switzerland.

CI, confidence interval. Mean physical activity per hour (for each hour of the day), for the total study population and its association with cardiovascular disease risk, using Cox-proportional hazard model, adjusted for age and sex.

### Supplemental Table 1 – Characteristics of included and excluded participants, survey 1 (Follow-up 2, 2014-17) and 2 (Follow-up 3, 2018-21), CoLaus|PsyCoLaus study, Lausanne, Switzerland

|  | **Survey 1** | |  | **Survey 2** | |  |
| --- | --- | --- | --- | --- | --- | --- |
|  | **Included** | **Excluded** | **P-value** | **Included** | **Excluded** | **P-value** |
| N | 2465 | 2416 |  | 1692 | 2059 |  |
| Female Sex, N (%) | 1362 (55.3) | 1327 (54.9) | 0.818 | 920 (54.4) | 1164 (56.5) | 0.185 |
| Age, mean (years) | 61.2 ± 9.7 | 64.7 ± 10.9 | <0.001 | 64.4 ± 9.5 | 65.9 ± 10.2 | <0.001 |
| Educational level, N (%) |  |  | <0.001 |  |  | <0.001 |
| Compulsory education | 353 (14.3) | 486 (20.1) |  | 214 (12.7) | 352 (17.1) |  |
| Apprenticeship | 883 (35.8) | 866 (35.9) |  | 586 (34.6) | 726 (35.3) |  |
| Secondary school | 282 (11.4) | 264 (10.9) |  | 197 (11.6) | 254 (12.4) |  |
| High school degree | 378 (15.3) | 334 (13.8) |  | 297 (17.6) | 277 (13.5) |  |
| University degree | 568 (23.1) | 463 (19.2) |  | 398 (23.5) | 448 (21.8) |  |
| Sleep chronotype, N (%) |  |  | <0.001 |  |  | <0.001 |
| Morning | 871 (35.3) | 579 (29.2) |  | 568 (33.6) | 478 (30.0) |  |
| Evening | 916 (37.2) | 577 (29.1) |  | 597 (35.3) | 481 (30.1) |  |
| Other | 678 (27.5) | 824 (41.6) |  | 527 (31.2) | 637 (39.9) |  |
| Alcohol consumption, N (%) |  |  | <0.001 |  |  | <0.001 |
| None | 551 (23.7) | 624 (32.6) |  | 550 (32.5) | 1142 (55.5) |  |
| 1-13/week | 1450 (62.3) | 1038 (54.3) |  | 935 (55.3) | 730 (35.5) |  |
| 14-27/week | 270 (11.6) | 203 (10.6) |  | 172 (10.2) | 148 (7.2) |  |
| 28+/week | 56 (2.4) | 48 (2.5) |  | 35 (2.1) | 39 (1.9) |  |
| Smoking status, N (%) |  |  | 0.086 |  |  | 0.050 |
| Never | 1052 (42.7) | 834 (41.1) |  | 761 (45.0) | 734 (43.1) |  |
| Former | 973 (39.5) | 781 (38.5) |  | 686 (40.5) | 670 (39.3) |  |
| Current | 440 (17.9) | 415 (20.4) |  | 245 (14.5) | 299 (17.6) |  |
| Body mass index (kg/m^2^) | 26.3 ± 4.6 | 26.6 ± 4.8 | 0.036 | 26.1 ± 4.5 | 26.5 ± 4.9 | 0.015 |
| Waist circumference (cm) | 91.2 ± 13.2 | 92.7 ± 13.9 | <0.001 | 91.3 ± 13.5 | 92.5 ± 14.0 | 0.013 |
| Diabetes, N (%) | 190 (7.7) | 308 (14.8) | <0.001 | 139 (8.2) | 244 (13.1) | <0.001 |
| Antidiabetic treatment, N (%) | 139 (5.6) | 258 (10.7) | <0.001 | 107 (6.3) | 189 (9.2) | 0.001 |
| Fasting glucose (mmol/l) | 5.4 ± 1.0 | 5.6 ± 1.2 | <0.001 | 5.5 ± 1.0 | 5.6 ± 1.2 | 0.013 |
| Fasting insulin (μIU/mL) | 7.5 [5.1 - 11.2] | 8.1 [5.5 - 12.8] | § <0.001 | 8.2 [5.7 - 12.4] | 8.8 [5.9 - 13.4] | § 0.001 |
| Lipid levels (mmol/L) |  |  |  |  |  |  |
| Total cholesterol | 5.4 ± 1.0 | 5.2 ± 1.1 | <0.001 | 5.3 ± 1.0 | 5.1 ± 1.1 | <0.001 |
| HDL cholesterol | 1.6 ± 0.5 | 1.6 ± 0.5 | 0.210 | 1.6 ± 0.4 | 1.6 ± 0.4 | 0.295 |
| LDL cholesterol | 3.2 ± 0.9 | 3.0 ± 1.0 | <0.001 | 3.1 ± 0.8 | 3.0 ± 0.9 | <0.001 |
| Triglycerides | 1.1 [0.8 - 1.5] | 1.1 [0.8 - 1.5] | § 0.013 | 1.1 [0.9 - 1.6] | 1.1 [0.8 - 1.6] | § 0.759 |
| Lipid-lowering medication, N (%) | 339 (14.2) | 600 (25.8) | <0.001 | 239 (47.6) | 396 (59.2) | <0.001 |
| Blood pressure (mmHg) |  |  |  |  |  |  |
| Systolic (mean, SD) | 126 ± 17 | 129 ± 19 | <0.001 | 128 ± 18 | 129 ± 19 | 0.008 |
| Diastolic (mean, SD) | 77 ± 11 | 77 ± 11 | 0.788 | 78 ± 10 | 78 ± 11 | 0.642 |
| Antihypertensive treatment, N (%) | 677 (27.5) | 1020 (42.2) | <0.001 | 522 (30.9) | 826 (40.1) | <0.001 |

Results are expressed as number of participants (column percentage) for categorical variables and as average ± standard deviation or median [interquartile range] for continuous variables. Between group comparisons performed using Pearson chi-square test for categorical variables and student’s t-test or Kruskal-Wallis test (§) for continuous variables. For excluded participants, the total number of participants may vary due to missing data.

### Supplemental Table 2 – Participant characteristics by PA timing clusters for the survey 1 and 2 (Follow-ups 2 and 3), CoLaus|PsyColaus study, Lausanne, Switzerland

|  |  | **Survey 1** | |  |  |  | **Survey 2** | |  |  |
| --- | --- | --- | --- | --- | --- | --- | --- | --- | --- | --- |
| **Cluster number** | **1** | **2** | **3** | **4** | **P-value** | **1** | **2** | **3** | **4** | **P-value** |
| N | 481 | 453 | 790 | 741 |  | 458 | 413 | 480 | 341 |  |
| Female Sex, N (%) | 261 (54.3) | 260 (57.4) | 500 (63.3) | 341 (46.0) | <0.001 | 262 (57.2) | 193 (46.7) | 307 (64.0) | 158 (46.3) | <0.001 |
| Age, mean (years) | 59.8 ± 9.4 | 64.2 ± 10.2 | 64.1 ± 9.3 | 57.1 ± 8.4 | <0.001 | 64.5 ± 8.6 | 62.6 ± 9.4 | 67.6 ± 9.2 | 62.0 ± 9.9 | <0.001 |
| Educational level, N (%) |  |  |  |  | <0.001 |  |  |  |  | <0.001 |
| Compulsory education | 66 (13.7) | 98 (21.6) | 129 (16.4) | 60 (8.1) |  | 54 (11.8) | 47 (11.4) | 82 (17.1) | 31 (9.1) |  |
| Apprenticeship | 142 (29.5) | 199 (43.9) | 292 (37.0) | 250 (33.7) |  | 131 (28.6) | 150 (36.3) | 211 (44.0) | 94 (27.6) |  |
| Secondary school | 71 (14.8) | 42 (9.3) | 77 (9.8) | 92 (12.4) |  | 67 (14.6) | 39 (9.4) | 43 (9.0) | 48 (14.1) |  |
| High-school degree | 74 (15.4) | 55 (12.1) | 138 (17.5) | 111 (15.0) |  | 90 (19.7) | 78 (18.9) | 67 (14.0) | 62 (18.2) |  |
| University degree | 128 (26.6) | 59 (13.0) | 153 (19.4) | 228 (30.8) |  | 116 (25.3) | 99 (24.0) | 77 (16.0) | 106 (31.1) |  |
| Body mass index (kg/m^2^) | 26.2 ± 4.9 | 26.4 ± 4.5 | 26.1 ± 4.5 | 26.5 ± 4.7 | 0.513 | 25.7 ± 4.0 | 25.9 ± 4.4 | 26.3 ± 4.6 | 26.9 ± 4.9 | 0.001 |
| Waist circumference (cm) | 90.9 ± 13.5 | 91.3 ± 13.1 | 90.7 ± 12.9 | 92 ± 13.3 | 0.293 | 89.6 ± 12.2 | 91.0 ± 13.6 | 91.3 ± 13.2 | 94.0 ± 15.0 | <0.001 |
| Sleep chronotype, N (%) |  |  |  |  | 0.293 |  |  |  |  | <0.001 |
| Morning person | 53 (11.0) | 259 (57.2) | 266 (33.7) | 293 (39.5) |  | 91 (19.9) | 217 (52.5) | 198 (41.3) | 62 (18.2) |  |
| Evening person | 308 (64.0) | 82 (18.1) | 305 (38.6) | 221 (29.8) |  | 210 (45.9) | 89 (21.6) | 113 (23.5) | 185 (54.3) |  |
| Other | 120 (25.0) | 112 (24.7) | 219 (27.7) | 227 (30.6) |  | 157 (34.3) | 107 (25.9) | 169 (35.2) | 94 (27.6) |  |
| Alcohol consumption, N (%) |  |  |  |  | 0.062 |  |  |  |  | 0.464 |
| None | 121 (26.5) | 122 (29.3) | 165 (22.0) | 143 (20.3) |  | 151 (33.0) | 124 (30.0) | 164 (34.2) | 111 (32.6) |  |
| 1-13/week | 270 (59.1) | 240 (57.7) | 484 (64.5) | 456 (64.9) |  | 248 (54.2) | 244 (59.1) | 262 (54.6) | 181 (53.1) |  |
| 14-27/week | 55 (12.0) | 43 (10.3) | 84 (11.2) | 88 (12.5) |  | 53 (11.6) | 38 (9.2) | 42 (8.8) | 39 (11.4) |  |
| 28+/week | 11 (2.4) | 11 (2.6) | 18 (2.4) | 16 (2.3) |  | 6 (1.3) | 7 (1.7) | 12 (2.5) | 10 (2.9) |  |
| Smoking status, N (%) |  |  |  |  | <0.001 |  |  |  |  | 0.018 |
| Never | 182 (37.8) | 192 (42.4) | 355 (44.9) | 323 (43.6) |  | 191 (41.7) | 183 (44.3) | 233 (48.5) | 154 (45.2) |  |
| Former | 182 (37.8) | 192 (42.4) | 323 (40.9) | 276 (37.3) |  | 206 (45.0) | 166 (40.2) | 192 (40.0) | 122 (35.8) |  |
| Current | 117 (24.3) | 69 (15.2) | 112 (14.2) | 142 (19.2) |  | 61 (13.3) | 64 (15.5) | 55 (11.5) | 65 (19.1) |  |

Results are expressed as number of participants (column percentage) for categorical variables and as average ± standard deviation for continuous variables. Between-group comparisons performed using chi-square for categorical variables and analysis of variance for continuous variables.

### Supplemental Table 3 – Participant characteristics by physical activity timing clusters based on METs, multivariable analysis, survey 1 (Follow-up 2, 2014-17), CoLaus|PsyCoLaus study, Lausanne, Switzerland

| **Cluster number** | **1** | **2** | **3** | **4** | **P-value §** |
| --- | --- | --- | --- | --- | --- |
| Hypertension | 1 (reference) | 1.20 (0.92 - 1.57) | 0.97 (0.75 - 1.26) | 1.13 (0.87 - 1.46) |  |
| Hypolipidemic drug treatment | 1 (reference) | 1.15 (0.81 - 1.63) | 0.91 (0.63 - 1.33) | 0.97 (0.68 - 1.38) |  |
| Diabetes | 1 (reference) | 1.48 (0.95 - 2.29) | 1.22 (0.74 - 1.99) | 0.90 (0.55 - 1.47) |  |
| Blood pressure (mm Hg) |  |  |  |  |  |
| Systolic | 126 ± 1 | 125 ± 1 | 124 ± 1 | 126 ± 1 | 0.281 |
| Diastolic | 77 ± 1 | 78 ± 1 | 77 ± 1 | 78 ± 1 | 0.561 |
| Lipid levels (mmol/L) |  |  |  |  |  |
| Total cholesterol | 5.51 ± 0.04 | 5.32 ± 0.04^a^ | 5.37 ± 0.04 | 5.40 ± 0.04 | 0.007 |
| HDL cholesterol | 1.61 ± 0.02 | 1.58 ± 0.02 | 1.59 ± 0.02 | 1.66 ± 0.02^b^ | 0.012 |
| LDL cholesterol | 3.31 ± 0.03 | 3.16 ± 0.04^a^ | 3.18 ± 0.04 | 3.19 ± 0.04 | 0.015 |
| Triglycerides | 1.31 ± 0.04 | 1.28 ± 0.05 | 1.31 ± 0.05 | 1.24 ± 0.05^c^ | 0.014 ‡ |
| Diabetes markers |  |  |  |  |  |
| Glucose (mmol/L) | 5.37 ± 0.03 | 5.34 ± 0.04 | 5.39 ± 0.04 | 5.43 ± 0.04 | 0.305 |
| Insulin (μIU/mL) | 9.1 ± 0.2 | 9.4 ± 0.2 | 9.0 ± 0.2 | 9.1 ± 0.2 | 0.373 ‡ |
| HbA_1_c (mmol/mol) | 37.5 ± 0.2 | 37.5 ± 0.2 | 37.7 ± 0.2 | 37.9 ± 0.2 | 0.536 |

§ for ANOVA; ‡ based on log-transformed data. Results are expressed as multivariable-adjusted odds ratio and (95% confidence interval) for hypertension, hypolipidemic drug treatment and diabetes, and as multivariable-adjusted marginal mean ± standard error for the other variables. Statistically significant (p<0.05) odds ratios are indicated in bold characters. Multivariable analysis performed using logistic regression for categorical variables and by ANOVA for continuous variables. For continuous variables, post-hoc bivariate comparisons were performed using Scheffe’s method: subscript a refers to comparison of clusters 2, 3 and 4 to cluster 1, subscript b to comparison of clusters 3 and 4 to cluster 2, and subscript c to comparison of cluster 4 to cluster 3; presence of the subscript indicates that clusters differ at p<0.05. All multivariable models adjusted for sex (male, female), age (continuous), educational level (basic, apprenticeship, secondary, high school, and university), alcohol consumption (none, 1-13, 14-27 and 27+ per week), smoking categories (never, former, current) and BMI categories (normal, overweight, obese). For blood pressure levels, a further adjustment on antihypertensive drug treatment (yes, no) was performed; for lipid levels, a further adjustment on hypolipidemic drug treatment (yes, no) was performed, and for diabetes markers, a further adjustment on antidiabetic drug treatment (yes, no) was performed.

### Supplemental Table 4 – Participant characteristics by physical activity timing clusters based on METs, multivariable analysis, survey 2 (Follow-up 3, 2018-21), CoLaus|PsyCoLaus study, Lausanne, Switzerland

| **Cluster number** | **1** | **2** | **3** | **4** | **P-value §** |
| --- | --- | --- | --- | --- | --- |
| Hypertension | 1 (reference) | 1.06 (0.79 - 1.42) | 1.34 (0.98 - 1.84) | **1.38 (1.01 - 1.91)** |  |
| Hypolipidemic drug treatment | 1 (reference) | 0.87 (0.51 - 1.49) | 1.30 (0.72 - 2.36) | 0.97 (0.53 - 1.75) |  |
| Diabetes | 1 (reference) | 0.90 (0.52 - 1.56) | 1.16 (0.67 - 2.01) | 1.51 (0.88 - 2.57) |  |
| Blood pressure (mm Hg) |  |  |  |  |  |
| Systolic | 127 ± 1 | 127 ± 1 | 129 ± 1 | 127 ± 1 | 0.310 |
| Diastolic | 77 ± 1 | 78 ± 1 | 78 ± 1 | 77 ± 1 | 0.991 |
| Lipid levels (mmol/L) |  |  |  |  |  |
| Total cholesterol | 5.43 ± 0.09 | 5.40 ± 0.07 | 5.27 ± 0.09 | 5.32 ± 0.09 | 0.507 |
| HDL cholesterol | 1.46 ± 0.03 | 1.54 ± 0.03 | 1.52 ± 0.03 | 1.44 ± 0.03 | 0.127 |
| LDL cholesterol | 3.23 ± 0.08 | 3.22 ± 0.06 | 3.13 ± 0.08 | 3.17 ± 0.08 | 0.743 |
| Triglycerides | 1.59 ± 0.09 | 1.40 ± 0.07 | 1.49 ± 0.09 | 1.56 ± 0.09 | 0.058‡ |
| Diabetes markers |  |  |  |  |  |
| Glucose (mmol/L) | 5.54 ± 0.04 | 5.46 ± 0.04 | 5.51 ± 0.04 | 5.55 ± 0.05 | 0.353 |
| Insulin (μIU/mL) | 10.3 ± 0.3 | 9.6 ± 0.3 | 9.7 ± 0.4 | 11.4 ± 0.4^b^ | 0.004‡ |
| HbA_1_c (mmol/mol) | 37.1 ± 0.3 | 37.0 ± 0.2 | 36.9 ± 0.3 | 37.4 ± 0.3 | 0.483 |

§ for ANOVA; ‡ based on log-transformed data. Results are expressed as multivariable-adjusted odds ratio and (95% confidence interval) for hypertension, hypolipidemic drug treatment and diabetes, and as multivariable-adjusted marginal mean ± standard error for the other variables. Statistically significant (p<0.05) odds ratios are indicated in bold characters. Multivariable analysis performed using logistic regression for categorical variables and by ANOVA for continuous variables. For continuous variables, post-hoc bivariate comparisons were performed using Scheffe’s method: subscript a refers to comparison of clusters 2, 3 and 4 to cluster 1, subscript b to comparison of clusters 3 and 4 to cluster 2, and subscript c to comparison of cluster 4 to cluster 3; presence of the subscript indicates that clusters differ at p<0.05. All multivariable models adjusted for sex (male, female), age (continuous), educational level (basic, apprenticeship, secondary, high school, and university), alcohol consumption (none, 1-13, 14-27 and 27+ per week), smoking categories (never, former, current) and BMI categories (normal, overweight, obese). For blood pressure levels, a further adjustment on antihypertensive drug treatment (yes, no) was performed; for lipid levels, a further adjustment on hypolipidemic drug treatment (yes, no) was performed, and for diabetes markers, a further adjustment on antidiabetic drug treatment (yes, no) was performed.

Supplemental Table 5 **–** Analysis of the associations between physical activity timing clusters and cardiovascular events, CoLaus|PsyCoLaus study, Lausanne, Switzerland, using inverse probability weighting to account for excluded participants.

| **Cluster number** | **1** | **2** | **3** | **4** |
| --- | --- | --- | --- | --- |
| Bivariate | 1 (reference) | **4.13 (1.36 - 12.6)** | **3.52 (1.20 - 10.3)** | 2.57 (0.84 - 7.81) |
| Multivariable 1 | 1 (reference) | **3.39 (1.09 - 10.5)** | 2.89 (0.99 - 8.47) | **3.21 (1.04 - 9.94)** |
| Multivariable 2 | 1 (reference) | **3.51 (1.12 - 11.0)** | **2.99 (1.02 - 8.76)** | **3.21 (1.04 - 9.96)** |

For bivariate and multivariable analysis, results are expressed as hazard ratio and (95% confidence interval). Analyses conducted using Cox regression. Multivariable model 1: adjusted for sex (male, female), age (continuous), educational level (basic, apprenticeship, secondary, high school, and university), alcohol consumption (none, 1-13, 14-27 and 27+ per week), smoking categories (never, former, current), and BMI categories (normal, overweight, obese). Multivariable model 2: as model 1, plus hypertension (yes, no), diabetes (yes, no) and hypolipidemic drug treatment (yes, no). Significant (p<0.05) coefficients are indicated in bold.

Supplemental Table 6 **–** Analysis of the associations between physical activity timing clusters and cardiovascular events, CoLaus|PsyCoLaus study, Lausanne, Switzerland, stratified by age or physical activity group.

| **Cluster** | **Age ^1^** | **Cases** | **Non-cases** | **HR (95% CI)** | **P-value** | **Interaction** |
| --- | --- | --- | --- | --- | --- | --- |
| Cluster 1 | <65 years | 2 | 283 | 1 |  |  |
|  | ≥65 years | 2 | 99 | 1 |  |  |
| Cluster 2 | <65 years | 8 | 185 | 6.37 (1.35 - 30.0) | 0.019 |  |
|  | ≥65 years | 11 | 160 | 3.35 (0.74 - 15.1) | 0.116 | 0.564 |
| Cluster 3 | <65 years | 11 | 350 | 4.82 (1.06 - 21.8) | 0.041 |  |
|  | ≥65 years | 19 | 290 | 3.20 (0.74 - 13.7) | 0.118 | 0.748 |
| Cluster 4 | <65 years | 10 | 538 | 2.46 (0.54 - 11.3) | 0.245 |  |
|  | ≥65 years | 7 | 84 | 4.49 (0.92 - 21.8) | 0.063 | 0.715 |
|  | **Physical activity ^2^** |  |  |  |  |  |
| Cluster 1 | Less active | 2 | 196 | 1 |  |  |
|  | More active | 2 | 186 | 1 |  |  |
| Cluster 2 | Less active | 14 | 140 | 7.20 (1.61 - 32.2) | 0.010 |  |
|  | More active | 5 | 205 | 1.62 (0.31 - 8.37) | 0.567 | 0.258 |
| Cluster 3 | Less active | 15 | 301 | 3.97 (0.90 - 17.5) | 0.068 |  |
|  | More active | 15 | 339 | 2.78 (0.63 - 12.3) | 0.176 | 0.866 |
| Cluster 4 | Less active | 11 | 281 | 4.36 (0.95 - 19.9) | 0.057 |  |
|  | More active | 6 | 341 | 1.88 (0.38 - 9.36) | 0.443 | 0.426 |

Results are expressed as hazard ratio and (95% confidence interval). Analyses conducted using Cox regression. ^1^ adjusted on sex; ^2^ adjusted on age and sex. Interaction : effect interaction between the less active / more active groups if p-value for interaction is <0.05.

### Annex 1- Adjudication procedure for cardiovascular disease events

Vital status of the participants was assessed at each follow-up exam. If the follow-up information letter was returned by the post office, or the participant did not respond to the successive phone call to schedule the follow-up exam, the civil register of the community in which the subject was living at the time of the last exam was contacted to determine the participant’s new address, or the occurrence and date of death. If the participant moved within Switzerland, the new address was systematically recorded, and a new contact was established. If no contact could be obtained, vital status was controlled through the civil register of their new community. If the participant moved outside Switzerland, no information regarding vital status could be obtained and the last contact date was used.

Cause of death was established using all available sources of information: general practitioners, any hospital in Switzerland (if death occurred in this setting), the pre-hospital emergency care unit of the City of Lausanne, the forensic medicine department of the University of Lausanne, and the Swiss Statistics of the Swiss Confederation. If medical records were available, cause of death was adjudicated by two internal medicine specialists. Official death certificates were used only if medical reports were unavailable.^1^

1. Beuret H, Hausler N, Nanchen D, Méan M, Marques-Vidal P, Vaucher J. Comparison of Swiss and European risk algorithms for cardiovascular prevention in Switzerland. *Eur J Prev Cardiol*. 2021;28(2):204-210. doi:10.1177/2047487320906305
